# Supplementary material for: A Likelihood Approach for Real-Time Calibration of Stochastic Compartmental Epidemic Models
Source: PLoS Comput Biol. 2017 Jan 17;13(1):e1005257. doi: 10.1371/journal.pcbi.1005257 (PMC5240920; doi:10.1371/journal.pcbi.1005257)
Supplement: S1 File — (TAR.GZ) [file pcbi.1005257.s014.tar.gz › HSPH_Online-SI-Revision/EnKF07/n1-extreme/n1-extreme_PF_EnKF-summery.pdf]

peak -8	R0	$1/\Theta_2$	inf	inflog	newly	newlylog	Reff	AttackRate
mean	35.7	52.9	1745.3	1.	7976.6	1.2	55.5	71.
median	37.1	51.1	1126.9	1.	2014.2	1.1	49.3	65.5
Quantile .1	24.5	39.9	197.7	0.4	413.6	0.5	39.2	37.5
Quantile .9	47.6	67.1	3180.1	1.4	11 024.3	1.8	75.8	113.
46	17	33						
length	50							

peak -4	R0	$1/\Theta_2$	inf	inflog	newly	newlylog	Reff	AttackRate
mean	31.1	59.	147.5	0.5	230.7	0.5	70.	99.4
median	29.6	60.2	94.2	0.5	204.6	0.5	70.3	99.5
Quantile .1	16.1	47.2	72.1	0.3	102.6	0.4	60.7	71.4
Quantile .9	50.9	69.6	303.6	0.8	436.	0.7	75.9	131.
42	9	3						
length	50							

peak	R0	$1/\Theta_2$	inf	inflog	newly	newlylog	Reff	AttackRate
mean	31.4	60.5	85.7	0.9	111.1	0.6	60.	114.3
median	27.5	60.1	86.	0.9	105.5	0.6	60.2	112.4
Quantile .1	17.1	48.4	79.3	0.6	83.5	0.5	50.7	90.7
Quantile .9	47.6	70.7	92.9	1.1	139.2	0.8	67.3	144.9
41	20	0						
length	50							

peak +4	R0	$1/\Theta_2$	inf	inflog	newly	newlylog	Reff	AttackRate
mean	32.8	60.9	91.4	0.7	145.9	0.5	66.8	105.6
median	30.3	62.3	86.8	0.7	129.2	0.5	58.9	107.7
Quantile .1	15.8	42.5	79.8	0.4	93.3	0.4	48.1	81.
Quantile .9	51.	74.1	105.6	0.9	224.9	0.6	91.9	131.8
43	9	0						
length	50							

peak +8	R0	$1/\Theta_2$	inf	inflog	newly	newlylog	Reff	AttackRate
mean	33.7	58.1	210.2	0.4	570.4	0.5	92.6	101.7
median	32.7	58.7	119.4	0.4	401.5	0.5	76.1	95.8
Quantile .1	12.6	39.9	77.7	0.3	189.5	0.4	48.5	82.3
Quantile .9	60.4	77.5	430.4	0.6	1029.1	0.7	145.5	129.4
39	17	0						
length	50							
